# Prevalence of HIV and hepatitis B coinfection in Ghana: a systematic review and meta-analysis

**DOI:** 10.1186/s12981-016-0107-x

**Published:** 2016-05-17

**Authors:** Akosua Adom Agyeman, Richard Ofori-Asenso

**Affiliations:** Research Unit, Health Policy Consult, P. O. Box WJ 537, Weija, Accra, Ghana

**Keywords:** HIV, Hepatitis B, Coinfection, Prevalence, Ghana, Meta-analysis

## Abstract

**Background:**

Human immunodeficiency virus (HIV) and hepatitis B virus (HBV) coinfection has been associated with higher morbidity and mortality and may impact significantly on healthcare resource utilization. However, in Ghana, accurate estimates of the prevalence of HIV/HBV coinfection needed to inform policy decisions and the design of public health interventions are currently lacking. In this study, our aim was to determine the HIV/HBV coinfection prevalence rate in Ghana.

**Methods:**

Primary studies reporting prevalence of HIV/HBV coinfection in Ghana were retrieved through searches conducted in PubMed, science direct, Google scholar and Africa journals online (AJOL) databases. The websites of the Ministry of Health and Ghana Health Service were also searched for related reports or reviews. Additionally, the online repository of two leading Ghanaian universities were searched to identify unpublished thesis related to the subject. All online searches were conducted between 01/03/2016 and 12/03/2016. Further searches were conducted through reference screening of retrieved papers.

**Results:**

Twelve (12) studies published between 1999 and 2016 and conducted across seven (7) regions of Ghana were included in this review. The three (3) regions with no studies’ representation were Upper East, Upper West and Central regions. The 12 included studies involved a total of 8162 HIV patients. The reported HIV/HBV coinfection prevalence rates ranged from 2.4 to 41.7 %. The pooled HIV/HBV coinfection prevalence rate was determined as 13.6 % (95 % CI 10.2–16.8 %; P < 0.001).

**Conclusions:**

In Ghana, about one in seven HIV patients may be also be chronically infected with HBV. Preventive interventions and strategic policy directions including systematic screening of all newly diagnosed HIV cases for coinfection will be needed, so as to improve management strategies for HBV infection and antiretroviral therapy (ART) implementation.

## Background

Human immunodeficiency virus (HIV) and hepatitis B virus (HBV) coinfection is common due to their shared transmission routes [1, 2]. Approximately, 10 % of all HIV infected patients worldwide are estimated to have chronic HBV coinfection [3]. However, wide regional variations are observed with coinfection prevalence rates estimated to be 5–10 % in areas such as North America, Europe and Australia compared to higher prevalence rates of 20–30 % in areas such as Sub-Saharan Africa and Asia [1, 2]. These statistics are of significant importance in Sub-Saharan Africa where over 70 % of the world’s 36.9 million people infected with HIV live [4].

Although, the specific mechanisms by which HBV interacts with HIV to influence disease progression are not clearly understood, HIV/HBV coinfection has been identified to facilitate higher levels of HBV replication, decreased rates of spontaneous resolution of the HBV infection, and higher risk of reactivation of previous infections [5–7]. Subsequently, HIV infected individuals have been found to be about six (6) times more likely to develop chronic HBV infection than their HIV negative counterparts [1, 8]. Additionally, the progression rate and complications such as liver fibrosis, cirrhosis, end-stage liver disease, hepatocellular carcinoma (HCC) and mortality due to liver pathology arising from HBV infection are accelerated in patients with HIV coinfection [3, 7]. In a recent synthesis of data from 12,382 patients in Greece for instance, there was a demonstrable significant 36 % increased rate of mortality attributable to the effect of HBV coinfection in HIV patients [9].

Antiretroviral therapy (ART) can be very challenging when coinfection is present as HIV-infected individuals are usually less responsive to treatments for HBV and have raised risk of hepatotoxicity and drug interactions [10, 11]. However, medications active against both HIV and HBV may allow for simplification of treatment regimen, although the overlapping antiviral spectrum of some HIV and HBV therapies can lead to additional complexities as a result of increased potential for the selection of drug-resistant mutations [10, 12].

In light of these background information, accurate estimates of the prevalence of HIV/HBV coinfection will be essential to inform evidence-based policy making (e.g. scaling up of screening programs) and resource allocation as well as impact positively on general prevention and treatment strategies for HBV/HIV coinfection especially regarding the use of highly active antiretroviral therapy (HAART) agents that also possess anti-HBV activity and its attendant implications [10].

A systematic review conducted by Barth et al. in 2010, estimated the overall prevalence of HIV/HBV coinfection in Sub Saharan Africa to be 15 % [13]. While this may be informative, it may not entirely represent the situation in Ghana as participants from studies conducted in Nigeria contributed more than two-fifth (40 %) of the overall population in that review [13]. Country-specific information regarding HIV/HBV coinfection prevalence that is relevant, defined and up to date may therefore serve greater purpose at informing specific prevention and treatment policies within Ghana. However, we have not found any published systematic review and meta-analysis specifically summarizing the prevalence of HIV/HBV coinfection in Ghana. We consider this to constitute an insufficient documentation of the country’s burden of HIV/HBV coinfection.

This study was therefore conducted to summarize the available information towards answering the key question; what is the prevalence of HIV/HBV coinfection in Ghana? This work was carried out as part of our series of research documenting the burden of viral hepatitis in Ghana.

## Methods

This review was conducted in accordance with the PRISMA (preferred reporting items for systematic reviews and meta-analyses) guidelines [14].

### Search strategy

To identify relevant studies, comprehensive searches were conducted by RO in PubMed, Science direct, Google SCHOLAR, and Africa journals online (AJOL) databases. The key words used were Hepatitis B, Hepatitis B surface antigen (HBsAg), HBV, HBV-DNA, human immunodeficiency virus, HIV, AIDS, coinfection, Prevalence and Ghana. In many instances, a combination of these keywords were explored (See "Appendix" section). AA also searched the digital institutional repository of the two leading Ghanaian universities, University of Ghana Legon (http://www.ugspace.ug.edu.gh/) and Kwame Nkrumah University of Science and Technology (http://www.ir.knust.edu.gh/) to identify unpublished thesis related to the subject. The websites of the Ministry of Health (http://www.moh-ghana.org/) and the Ghana Health Service (http://www.ghanahealthservice.org/) were also searched for related reports and reviews using selected phrases such as ‘Hepatitis B and HIV coinfection’. Online searches were conducted between 01/03/2016 and 12/03/2016. All references in selected articles were further screened for additional publications.

### Inclusion and exclusion of studies

Studies were included only if they reported chronic HBV infection prevalence rate among HIV-infected persons in Ghana. As generally recommended, laboratory diagnosis of chronic HBV infection focuses on the detection of hepatitis B surface antigen (HBsAg) [15]. Hence, only studies reporting prevalence of HBV in HIV persons based on HBsAg seropositivity were included. Studies reporting HBV prevalence in general populations (HIV negative cohorts) or those presenting HIV/hepatitis C (HCV) coinfection prevalence were excluded. Only studies published in English were selected. For duplicate studies, the version published first or one with complete dataset were included.

### Quality assessment and data extraction

Studies’ qualities were assessed using a 12-point scoring system based on the Downs and Black checklist as adopted in similar reviews [10, 16, 17]. These were: (objective of the study clearly described, study design clearly stated, participants representative of the population from which they were recruited, participants accrued during the same time period, modest sample size, management of missing data, age, gender and other characteristics explored/reported, e.g. were confounders reported, was detection method of HBV reported, were potential biases reported, was outcome clearly described?), the assessment also included other items known to be associated with study quality [10, 17]. Each study was issued with a unique number for identification purposes and the following descriptive information collected; author details, year of publication, region of Ghana, type of study population, mean age of subjects, number of subjects involved (sample size), setting (rural vs urban), gender of study participants and the HIV/HBV coinfection prevalence rate. Data were independently extracted by RO and AA and compared. Any disagreements or discrepancies were resolved by consensus-based discussions.

### Data analysis

We analyzed the results by meta-analysis proportions performed with OpenMeta (analyst) software, an open-source, cross-platform software for advanced meta-analysis [18] and StatsDirect statistical software (Version 3.0.0, StatsDirect Ltd, Cheshire UK) [19]. Individual study proportions were assessed at 95 % confidence interval (CI) as well as the pooled effect. Between-study heterogeneity was assessed by the Quoran (Q) statistic test and the *I*^2^ statistic, which represents the percentage of total variation across studies, attributable to heterogeneity rather than to chance [20]. A *p* value of <0.1 was considered to be statistically significant for the Q-statistics test and an I^2^ >50 % was deemed to represent meaningful heterogeneity in which case the random effect model (DerSimonian-Laird) was adopted over fixed effect model in the summary of pooled analysis [20]. To assess the publication bias and small-study bias, a funnel plot of the data was applied. In addition, Egger and Begg’s tests were used to detect publication bias [21, 22]. A leave-one-out sensitivity analysis was performed by iteratively removing one study at a time to confirm how each individual study affects the overall estimate of the rest of the studies [23]. For all computations except the between-study heterogeneity testing, statistical significance was set at p < 0.05.

### Ethical approval

An ethical approval was not required for this study as it was based on data/information retrieved from published studies already available in the public domain.

## Results

### Overview of studies

**Fig. 1:**
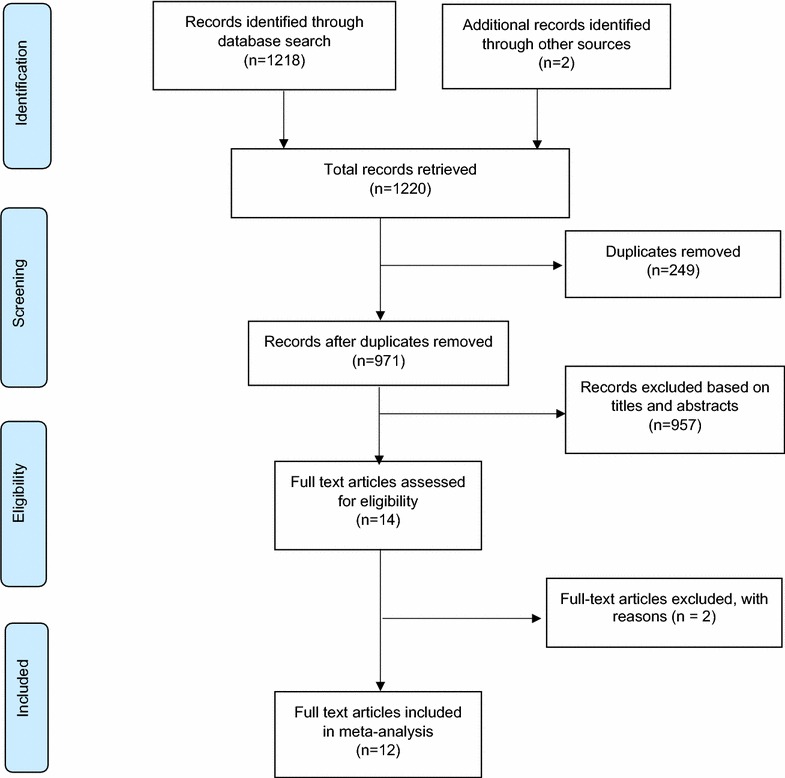
A schematic flow of studies’ search and retrieval processes

Figure 1 outlines the articles’ search and retrieval steps. A total of 1220 citations were identified through electronic search and other sources. After the exclusion of duplicates and irrelevant studies based on titles and abstracts, fourteen (14) articles were retrieved for detailed full-text analysis. Out of the 14 studies, twelve (12) met the inclusion criteria for addition to the review [24–35]. The 12 studies (Table 1) reported HBV coinfection prevalence rate in a total HIV-positive patient’s population size of 8162. The sample size (number of HIV infected patients) across the 12 studies ranged from 12 to 3108. The studies were conducted across seven (7) of the ten (10) regions of Ghana. The regional breakdown of the studies were as follows; Ashanti (n = 3), Brong-Ahafo (1), Eastern (2), Western (1), Northern (1), Greater Accra (3) and one study that involved multiple regions (Greater Accra, Ashanti, Eastern and Volta). Fifty-eight percent (7/12) of studies were conducted among HIV patients visiting HIV clinics whereas 17 % (2/12) were conducted among blood donors with same proportion (17 %, 2/12) of studies being carried out in pregnant women attending antenatal clinics. The oldest identified study was published in 1999 [24] and the most recent study identified was published in 2016 [35]. Ninety-two percent (92 %) of studies were published within the last decade (2006–2016) and as much as seventy-five percent (75 %) of studies were published within the last five (5) years (2011–2016). In studies that presented gender distribution, female participants were of higher proportion. The overall quality grading identified 59, 33 and 8 % of studies included in the review to be of high, moderate and low quality respectively.

**Table 1:** Descriptive characteristics of studies

Study no	Author details	Year of publication	Design	Region of study	Study population	Mean age of participants (years)	Sample size (no. of HIV + individuals)	Prevalence of HBV coinfection (%)	Gender (female, %)	Quality grade
1.	Brandful et al. [24]	1999	Cross-sectional	Greater Accra, Ashanti, Eastern and Volta	STD and general outpatient patients	34	182	16.5	44	High
2.	Apea-Kubi et al. [25]	2006	Prospective observational study	Greater Accra	Pregnant women and gynecological patients	29.6	12	41.7	100	High
3.	Geretti et al. [26]	2010	Cross-sectional	Ashanti	HIV clinic	NS	838	16.7	NS	High
4.	Cho et al. [27]	2012	Cross-sectional	Eastern	Pregnant women	NS	75	18.7	100	Medium
5.	Kubio et al. [28]	2012	Report review	Northern	Blood donors	NS	33	30.3	NS	Low
6.	Kye-Duodu [29]	2012	Cross-sectional	Eastern	HIV clinic	40.8	320	8.8	67.9	Medium
7.	Sagoe et al. [30]	2012	Cross-sectional	Greater Accra	HIV clinic	≥18	138	13.0	71 %	High
8.	Walana et al. [31]	2014	Cross-sectional	Brong-Ahafo	Blood donors	NS	168	2.4	NS	Medium
9.	Anyimah [32]	2015	Cross-sectional	Western	HIV clinic	38	125	17.6	68.8	Medium
10.	King et al. [33]	2015	Cross-sectional	Ashanti	HIV clinic	NS	1520	15.5	NS	High
11.	Stockdale et al. [34]	2015	Cross-sectional	Ashanti	HIV clinic	40	1643	14.0	58.5	High
12.	Archampong et al. [35]	2016	Cross-sectional	Greater Accra	HIV clinic	NS	3108	8.3	59.1	High

*NS* not specified, *STD* sexually transmitted diseases, *HIV* human immunodeficiency virus

### Meta-analysis findings

**Fig. 2:**
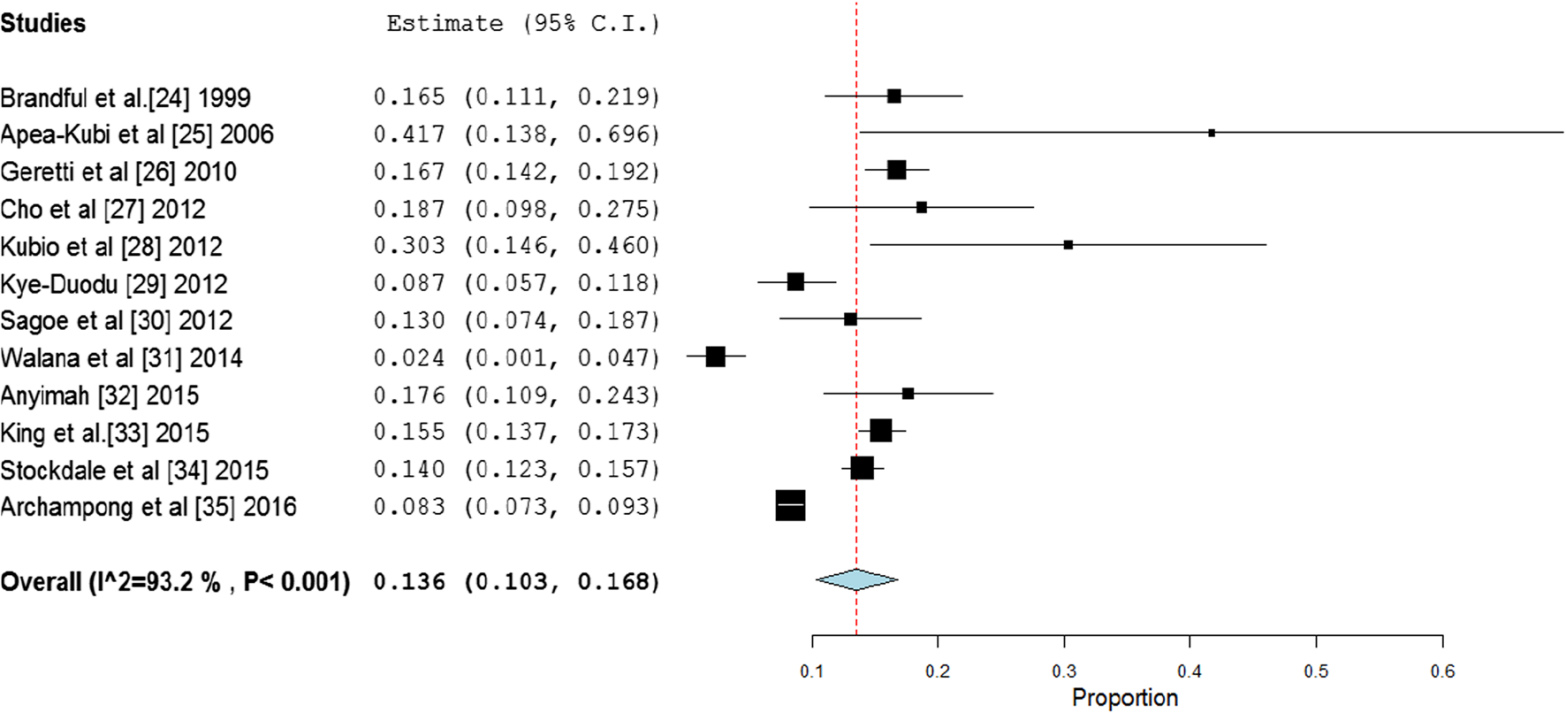
Forest *plot* of reported HIV/HBV coinfection prevalence rates across studies conducted in Ghana between 1999 and 2016

A total of 8162 HIV infected patients were involved in the 12 studies included in this review. The pooled HIV/HBV coinfection prevalence rate (Fig. 2) across the twelve (12) studies published between 1999 and 2016 in Ghana was 13.6 % (95 % CI 10.3–16.8 %; p < 0.001). Heterogeneity Chi squared (Q) was 161.7 (degree of freedom, *d.f* = 11), and I^2^ was determined as 93.2 % for the degree of inconsistency. The estimate of between-study variance Tau-squared was 0.002.

**Fig. 3:**
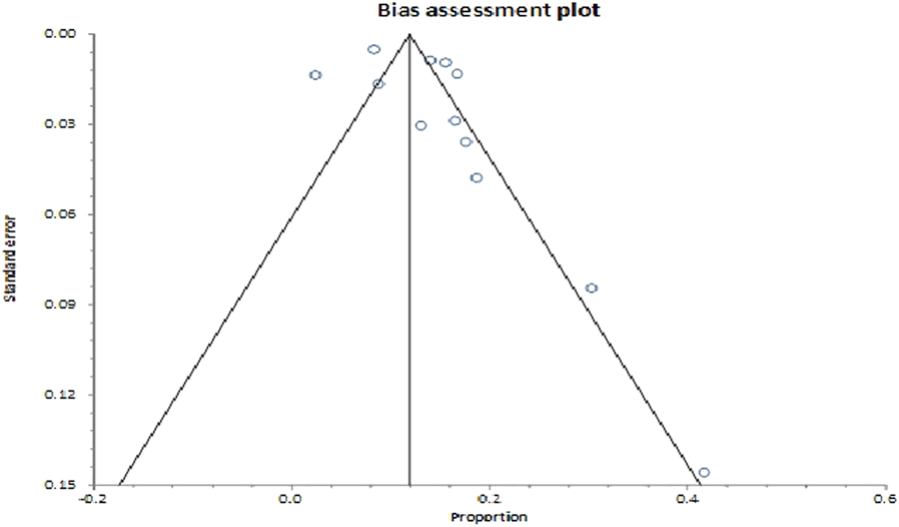
Bias assessment *plot* of reported HIV/HBV coinfection prevalence rates across studies published in Ghana between 1999 and 2016

A funnel plot of HBV/HIV coinfection prevalence rates did not reveal a completely symmetrical display of the prevalence rates reported by the various studies (Fig. 3). However, we did not deduce any strong evidence of publication bias as revealed by Egger’s (p = 0.1604) and Begg’s (p = 0.7373) tests.

**Fig. 4:**
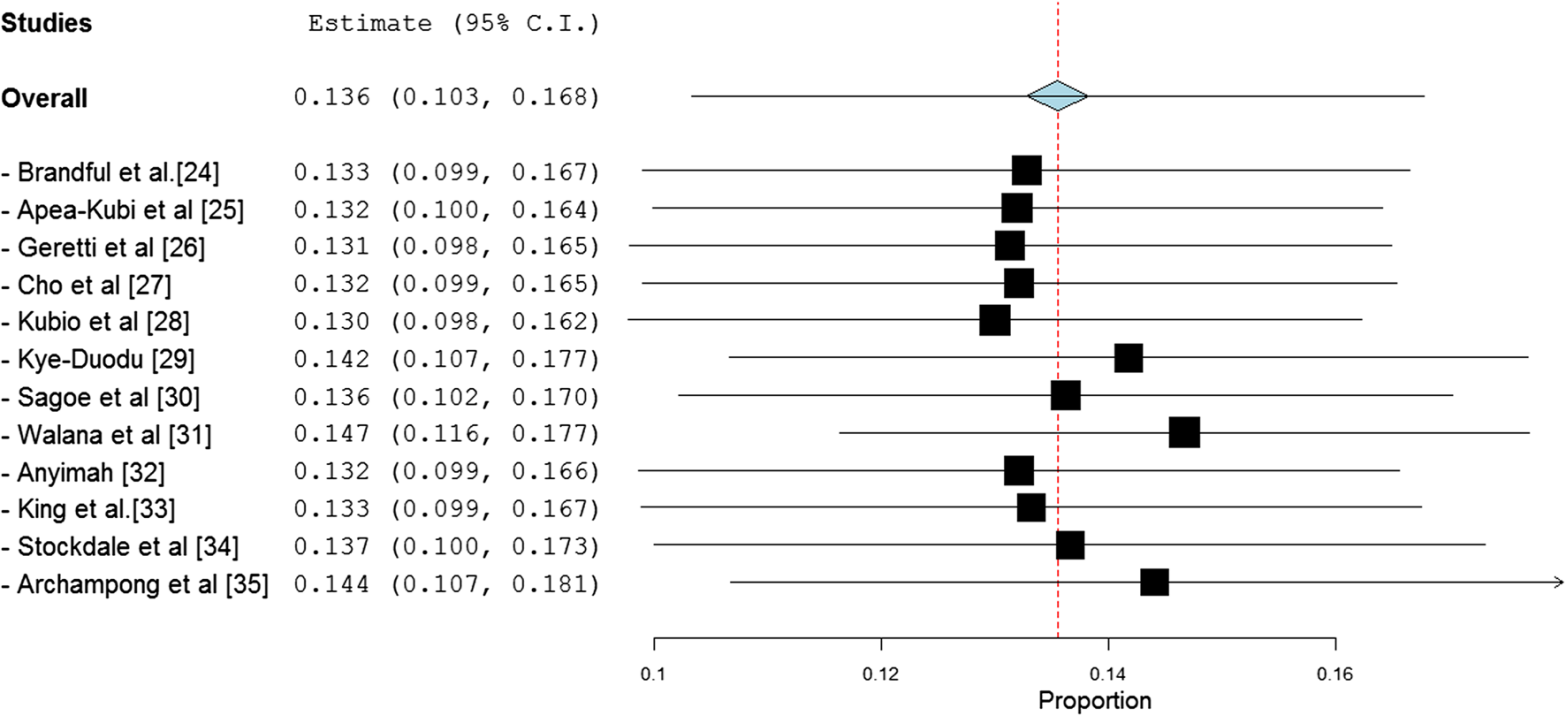
A leave-one-out forest *plot* of HIV/HBV coinfection prevalence rates across studies published in Ghana between 1999 ad 2016

To assess the robustness of the HIV/HBV coinfection prevalence results, we performed a leave-one-out sensitivity analysis by iteratively removing one study at a time while recalculating the coinfection prevalence rate. The weight of the individual summaries on the pooled effect showed that the prevalence estimate was dominated by Walana et al. [31], Archampong et al. [35] and Kye-Duodu [29] (Fig. 4).

## Discussion

The overall prevalence of HIV/HBV coinfection was determined to be high at 13.6 %, suggesting that about one in seven HIV positive patients in Ghana may be concurrently suffering from chronic HBV infection. Even for the analysis presented in this review, it is likely that the pooled estimates may be modest considering that majority of studies failed to account for the presence of occult HBV which is described as the occurrence of replication-competent HBV DNA in the liver with undetectable hepatitis B surface antigen (HBsAg) [36]. This often occurs after progressive disappearance of HBsAg in the years following infection and persisting in low-level carriers [37].

A recent systematic review estimated the prevalence of chronic HBV infection among Ghanaians to be 12.3 % [16], suggesting that HIV patients in Ghana tend to develop chronic HBV infection more so than the general population. Similar trends have been observed across many parts of the world and there are theoretical explanations to account for this, including the shared transmission routes of both diseases and the limited immune system capacity to resolve acute HBV infections in HIV patients [2, 3].

In Ghana, HBV testing and monitoring in HIV patients is not routine. As a result, although, the World Health Organization (WHO) recommends that ART be initiated in HIV co-infected patients irrespective of CD4 count [11], this is practically not being followed. The consequences has been that several HIV/HBV co-infected patients do not benefit from early treatment programs since current practices only takes into consideration the patient’s CD4 count level for the purpose of initiating ART [11]. The overall high prevalence of HBV in HIV patients as determined in this review necessitates the need for a national policy to offer HBV screening as part of the comprehensive care for all HIV positive persons as done in advanced countries like US and Canada where coinfection rates are even lower [2]. HIV patients who are detected to be HBV-seronegative, should be offered vaccination which ideally should be covered under the country’s National Health Insurance Scheme (NHIS) to ensure that most patients can benefit.

HIV/HBV coinfection increases the morbidity and mortality beyond those individually caused by both infections and this is likely to impose significant constraints on the already stretched health resources in Ghana. In HIV patients, the concurrent infection with HBV has been recognized to lead to increased tendency for the occurrence of AIDS-related and non-AIDS-related clinical outcomes, such as end-stage liver diseases including cirrhosis and HCC [38, 39]. Already, HBV is a significant contributor to the burden of HCC in Ghana. In a study by Blankson et al., at least two in five cirrhotic patients in Ghana were identified to be suffering from chronic HBV infection [40]. High rates of coinfection are likely to exacerbate these statistics.

The treatment for chronic HBV in Ghana is estimated to cost about $100–150 a month or same weekly to take an injection for 48 weeks as a way of managing the condition [16]. These costs are likely to be beyond the affordability of most HIV patients in Ghana neither will it be sustainable to finance through any public health funding scheme. Primary prevention through measures such as vaccination therefore seems the most reasonable approach in these circumstances. Such measures must be accompanied by widespread education campaigns regarding the transmission and infection dynamics of these viral pathogens. In Ghana, knowledge and awareness on HBV is documented to be low [41, 42]. In a recent assessment of 200 certified barbershops within the Kumasi metropolis in the Ashanti region, less than 10 % knew the route of transmission of HBV [41]. Additionally, the broader societal misconceptions regarding HBV such as the conceptualization of the disease as a purely sexually transmitted infection should be addressed through effective public education [16].

The high HIV/HBV coinfection rate should be given the needed attention and addressed to avert undue consequences. The economic and health-related (arising from ARTs, management of opportunistic infections etc.) cost of HIV alone is enormous and likely to be exacerbated by concurrent infections with HBV. Moreover, as has been widely observed, peak ages for AIDS cases in Ghana are 25–34 years for females and 30–39 years for males [43]. These age groups represent some of the most productive years and therefore increased morbidity and mortality arising from coinfection with HBV is likely to result in significant loss of man power and thereby impact on economic development in the country.

There are some limitations to this study. The accuracy of detection of active HBV infection depends on a number of factors such as the screening method employed [16]. Over the years, the sensitivity and specificity of HBV screening tools have improved and this could impact on the difference prevalence rates reported across studies published in different years. While all efforts were made to explore all resources, we retrieved only studies from seven of the 10 regions of Ghana. The overall estimate may therefore not fully represent the national situation. However, it is worth mentioning that according to the Ghana AIDs Commission [44], over 80 % of HIV patients in Ghana, reside in these seven region and the overall estimate presented in this review may therefore not deviate significantly from a true national prevalence. Nevertheless, this does not eliminate the fact that only a large representative national epidemiological study conducted at the same time in all regions can give a more reliable and accurate overall prevalence of HBV/HIV coinfection in Ghana [10]. This will also help to address the high heterogeneity as observed in this review.

Estimates of HIV/HBV coinfection prevalence rate in Ghana are currently lacking and as such this study opens new doors towards building a stronger evidence regarding HIV/HBV burden in Ghana. The HIV/HBV coinfection prevalence reported in this review should guide policy makers and health personnel towards improving the care of HIV/AIDS and HIV/HVB co-infected individuals.

## Conclusion

The prevalence of HIV/HBV coinfection in Ghana is high. Preventive interventions and strategic policy directions including systematic screening of all newly diagnosed HIV cases for coinfection will be needed, so as to improve management strategies for HBV infection and antiretroviral therapy (ART) implementation.

## Appendix

See Table 2.

**Table 2:** Database search strategy

Steps	Keywords	Result
I.	Human immunodeficiency virus or HIV or AIDS or acquired immune deficiency syndrome	#1
II.	Hepatitis B or HBV or HBsAg or HBV-DNA or hepatitis B surface antigen	#2
III.	Ghana	#3
IV.	Prevalence or seroprevalence	#4
V.	#1 and #2 and #3 and #4	Search results

## Abbreviations

AIDS: acquired immune deficiency syndrome
ART: antiretroviral therapy
HBV: hepatitis B virus
HBsAg: hepatitis B surface antigen
HCC: hepatocellular carcinoma
HIV: human immunodeficiency virus
NHIS: National Health Insurance Scheme
PRISMA: preferred reporting items for systematic reviews and meta-analyses
SSA: Sub-Saharan Africa
WHO: World Health Organization
